# Vitamin D Receptor *FokI, ApaI*, and *TaqI* Polymorphisms in Lead Exposed Subjects From Saudi Arabia

**DOI:** 10.3389/fgene.2019.00388

**Published:** 2019-04-26

**Authors:** Abjal Pasha Shaik, Abbas H. Alsaeed, M. Faiyaz-ul-Haque, Mikqdad A. Alsaeed, Abdullah A. Alyousef, Vamsee K. Bammidi, Asma Sultana Shaik

**Affiliations:** ^1^Department of Clinical Laboratory Sciences, King Saud University, Riyadh, Saudi Arabia; ^2^Department of Pathology, College of Medicine, King Saud University, Riyadh, Saudi Arabia; ^3^College of Medicine, King Saud bin Abdulaziz University for Health Sciences, Riyadh, Saudi Arabia; ^4^The Unicare Group, Burton-on-Trent, United Kingdom

**Keywords:** VDR gene polymorphisms, FokI, ApaI, TaqI, lead exposure, Saudi Arabia

## Abstract

Vitamin D receptor (VDR) gene polymorphisms were reported to influence blood lead levels (BLL) and the response of subjects to the symptoms of lead toxicity. However, no studies have been conducted in the Saudi Arabian population which has unique ethnicity and socio-demographic features. This study examined the polymorphisms in exon 2 (allele 1) and intron 8 (allele 2 and allele 3) of VDR gene and their relation to BLLs. As per the CDC guidelines, the recruited lead-exposed workers (*N* = 130) were categorized to two groups viz., low BLL group (<10 μg/dL) and high BLL group (>10 μg/dL). The low BLL group had a mean BLL of 4.37 μg/dL, while the high BLL group had levels of 18.12 μg/dL (*p* < 0.001). Overall, the genetic variants, TC and CC in the VDR FokI were significantly associated with a risk of lead toxicity and the allele “C” was a risk factor (*p* = 0.00026). Furthermore, the TT genotype of VDR ApaI significantly increased the risk of developing lead poisoning (*p* = 0.0006). The VDR TaqI SNP was not significantly associated with lead toxicity. The highest BLLs for VDR FokI-CC, VDR ApaI-GG, and VDR TaqI-TT genotypes from High BLL group were 18.42, 15.26, and 18.75 μg/dL, respectively. Older age (51–60 years) was found to be a significant confounding factor for BLLs (*p* = 0.012). Additional studies in larger sample sizes are needed to firmly establish the role of VDR genotypes and genetic susceptibility to lead poisoning.

## Introduction

The heavy metal lead (Pb) is used in battery manufacturing, smelting, mining, and various other occupations (ChemIDplus, 2005) including non-occupational uses in traditional folk medicine across Saudi Arabia. Therefore, lead poisoning can occur through a variety of routes such as ingestion, inhalation, or through the skin (i.e., transdermally) (Bellinger, 2004; Agency for Toxic Substances and Disease Registry [ATSDR], 2006; HSDB, 2007; Registry of Toxic Effects on Chemical Substances [RTECS], 2007). The Centers for Disease Control and Prevention (CDC, 1991) defines a concentration of more than 10 μg/dL as elevated and emphasizes the need for clinical intervention. Taken together, it is evident that any xenobiotic exposure can pose serious health concerns and biomonitoring of exposed subjects through toxicogenetic approaches are increasingly being recommended (Registry of Toxic Effects on Chemical Substances [RTECS], 2007).

The vitamin D receptor (VDR) gene that encodes the nuclear hormone receptor for vitamin D_3_ (Rezende et al., 2008) is being extensively investigated for its toxicogenetic role in Pb toxicity. VDR is principally involved in mineral metabolism, although the receptor regulates a variety of other metabolic pathways involved in immune response and cancer. VDR is expressed in the intestine, thyroid and kidney, and has a vital role in calcium homeostasis. The human gene encoding the VDR has been localized at chromosome 12q12-q14 (Rezende et al., 2008). Vitamin D is a key regulator of calcium uptake in the gut, and feeding studies in animals report increased lead concentrations in the kidney and bone tissues among animals fed with higher concentrations of vitamin D (Ross et al., 2011). The VDR gene regulates the production of calcium-binding proteins and accounts for up to 75% of the total genetic effects on bone density (He et al., 2015). This is consistent with the hypothesis that calcium levels reduce lead uptake, although, studies have identified an inverse correlation between low bone mineral density and whole blood lead concentration (HSDB, 2007; Xue and Fleet, 2008; Fleet and Schoch, 2010). Therefore, genetic polymorphisms in genes related to calcium uptake may consequently influence uptake of lead. Polymorphisms of the gene encoding for the VDR gene are of interest in lead-exposed populations because of the importance of the receptor for calcium absorption and bone mineralization (Wananukul et al., 2012; Moran et al., 2014).

Owing to fact that calcium and lead are divalent cations, they often can participate in the same molecular mechanisms and can be transported into blood, and to the bone. Furthermore, the various polymorphisms in the VDR gene may influence bone density. Based on restriction fragment length polymorphism (RFLP) analyses using Taq I, Fok I, and Bsm I restriction enzymes, 3 genotypes of VDR have been identified which are correlated with bone mineral density and bone mineralization (Onalaja and Claudio, 2000). The cell and molecular mechanisms of divalent calcium ions can be mimicked by divalent lead ions which can influence the levels of accumulation of lead in tissues. These pathways affect lead absorption from the gastrointestinal tract and may affect lead storage and/or release from bone. The VDR gene, thus, influences the toxicokinetics of lead concentrations but such studies with relation to BLL have not been investigated in Saudi Arabia so far.

Since VDR plays an important role in lead absorption, defining individuals susceptible to the toxic effects of lead was deemed necessary more so due to the heterogeneity of the Arab population which has unique ethnicity and socio-demographic features. In addition, such studies in the Saudi Arabian population will help understand the role of VDR gene in lead toxicity. The current study evaluated the influence of VDR gene polymorphisms in subjects occupationally exposed to lead with relation to BLLs.

## Materials and Methods

This was an observational cross-sectional epidemiological study conducted between February 2015 – February 2017). Subjects from Riyadh region, Saudi Arabia who were working in battery manufacturing, painting and automobile repair shops, and plumbers for at least 1 year prior to the study were included. The details of disease history, dietary habits, job, income, education, drug usage, use of vitamins and/or antioxidants, smoking, and other habits were collected from all subjects. The study was approved by Institutional Ethics Review Board (15/0163/IRB, College of Medicine and King Khalid University Hospital, King Saud University, Riyadh, KSA) and all subjects provided signed informed consent before enrolment into the study. A comprehensive clinical assessment of hematological and neurological diseases was performed on all subjects. Patients using antioxidants/vitamin supplements or other drugs, radiotherapy, and substance abuse were excluded from the study. Blood samples were collected from all subjects. The BLLs were measured using LeadCare II analyzer as per the manufacturer’s instructions (Magellan Diagnostics, Meridian Bioscience, OH, United States). In the present study, the effect of three SNPs (FokI, ApaI, and TaqI polymorphisms) in the *VDR* gene was evaluated using polymerase chain reaction (PCR). The details of primers along with fragment sizes and their allelic variants upon RFLP are presented as Supplementary Material.

### Genetic Analysis

Genomic DNA was extracted using Qiagen QIAamp DNA Blood Mini Kit from whole blood samples stored in EDTA coated tubes. The concentration of genomic DNA was determined by quantitative method, based on optical density measurement. DNA was quantified using NanoDrop UV/Vis Spectrophotometer (Thermo Scientific, United Kingdom). The purity of DNA was determined by calculating the ratio of absorbance at 260 nm to absorbance at 280 nm (A_260_/A_280_). Pure DNA should have an A_260_/A_280_ ratio of 1.7 – 1.9, respectively.

### Amplification for SNPs

Genotyping was carried out using the PCR-RFLP. PCR amplification products were obtained in a Thermal cycler (Mastercycle personal, Eppendorf, Germany) using the QIAGEN HotStarTaq Master Mix Kit in a final volume of 50 μl [2 μl genomic DNA (2 μg/μl), 25 μl HotStarTaq Master Mix, 19 μl RNase free water, and 2 μl of each primer]. The amplification conditions were as follows: initial denaturation at 95°C for 15 min, and 35 cycles of denaturation at 94°C for 1 min, annealing at 55°C for 1 min and extension at 72°C for 1 min, followed by final extension at 72°C for 10 min. The PCR products were purified using the Bioline Inc., United States kit. The DNA bands were visualized under UV light and photographed using gel documentation software (Molecular Imager^®^ Gel Doc^TM^ XR + Systems with Image Lab^TM^ 2.0 Software, BioRad, United States). The resulting DNA fragments were subjected to restriction digestion using respective enzymes. The Eppendorf tubes for RFLP were prepared as follows: 10 μl of PCR product, 16.3 μl of sterile, deionized water, 0.2 μl of 100X BSA, 2 μl of 10X RE Buffer, and mixed by pipetting. Finally, 1 μl each of the respective restriction enzymes were added. The tubes were incubated (2 h at 65°C for BsmI; 2 h at 37°C for ApaI; and 3 h at 55°C for Fok I polymorphisms) and heat inactivated for 15 min at 65–67°C. The genotypes were resolved on 2% (w/v) agarose gels.

### Statistical Analysis

The primary objective was to evaluate if subjects with polymorphisms in certain SNPs at VDR gene may be directly associated with BLLs. The overall sample size was calculated after adjusting for multiplicity and based on 5% significance level. A minimum sample size of 40 subjects was needed to detect an association. Assuming for a drop-out rate and/or ineligibility of approximately 20%, an evaluable sample size of 127 subjects required 159 subjects to be recruited. Unless specified, data are presented as mean ± SE. A *p* < 0.05 was considered statistically significant. Chi-square analysis was used to check for distribution of genotypes (in Hardy–Weinberg equilibrium) and alleles.

A multiple regression model was employed to assess the relationship between confounding factors such as age, while correcting for other determinants including type of work (radiator repair, exhaust repair, welding, mechanical repair, painting, and plumbing), years of exposure, education status, disease status, and income group. Since most subjects were non-smokers, non-obese, and consumed non-vegetarian meals, these parameters could not be taken into account during analyses. Statistics was calculated using SPSS software and correction for sample sizes and confounding factors was performed before interpretation of results.

## Results

The current study included male subjects aged (19–65 years). Some subjects did not participate in the clinical investigation or interview procedure and thus did not provide signed informed consent. Subjects were classified as per CDC guidelines as having BLLs of either >10 μg/dL (High BLL group; *N* = 22) or <10 μg/dL (Low BLL group; *N* = 108). Subjects with very low, uninterpretable or uncertain BLLs were excluded from analyses. The mean age of all subjects was 32.9 to 35.8 years.

The mean BLLs in low BLL group was 4.37 μg/dL while the subjects from high BLL group had mean BLLs of 18.12 μg/dL (*p* < 0.001). BLLs of <3.3 μg/dL were found among subjects who worked in the same industry but in different roles such as administration, drivers, and security personnel.

The distribution of genotypes of VDR FokI in the low BLL group deviated from the Hardy–Weinberg equilibrium (Table 1). The allele and genotype frequencies of the VDR SNPs FokI, ApaI, and TaqI in the low BLL and high BLL groups are presented in Table 2. The frequency of VDR FokI TT, TC and CC and VDR ApaI GG, GT and TT genotypes were 30.2, 34.4, and 27.7% and 40.9, 41.8, and 17.2%, respectively. For the VDR TaqI polymorphism, the TT, TC and CC genotypes were 36.6, 42.8, and 20.5% in the studied population. Overall, the genetic variants, TC and CC in the VDR FokI were found to be significantly associated with a risk of developing lead poisoning with the minor allele “C” being a risk factor (*p* = 0.00026). Although the GT genotype of VDR ApaI tended to be a risk factor, the association was not statistically significant. Moreover, the TT genotype of VDR ApaI conferred about three-fold significantly increased risk of developing lead poisoning compared to the GG genotype (OR = 9.641, χ^2^ = 11.64, *p* = 0.0006) (Table 2). In this case again, the minor “T” allele was found to be a risk factor for lead toxicity (*p* = 0.00051). None of the genotypes/alleles of the VDR TaqI SNP were significantly associated with lead toxicity, although the CC genotype and “C” allele indicated a probable role (Table 2). Irrespective of the genotypes, all subjects from high BLL group had significantly increased BLLs compared with normal group. The results are presented in Table 3.

**Table 1:** The distribution of genotypes and allele frequencies in low vs high BLL subjects.

SNP	Genotype	Low BLL group (frequency)	HWE *P*-value	High BLL group (frequency)	HWE *P*-value
VDR FokI	TT	36 (0.521)	0.001^∗∗∗^	0 (0.27)	0.3
	TC	34 (0.428)		7 (0.60)	
	CC	31 (0.05)		11 (0.13)	
	**Allele**				
	T	0.52		0.19	
	C	0.48		0.81	
VDR ApaI	GG	47 (0.271)	0.45	3 (0.33)	0.85
	GT	42 (0.485)		9 (0.39)	
	TT	13 (0.242)		8 (0.25)	
	**Allele**				
	G	0.67		0.38	
	T	0.33		0.62	
VDR TaqI	TT	37 (0.014)	0.2	4 (0.03)	0.96
	TC	38 (0.242)		10 (0.11)	
	CC	17 (0.742)		6 (0.86)	
	**Allele**				
	T	0.61		0.45	
	C	0.39		0.55	


*^∗∗∗^*p* < 0.001.*

**Table 2:** Distribution of VDR SNPs genotype and allele frequencies in the overall study population.

SNP	Genotype	OR (95% CI)	χ2-Value	*P*-Value
VDR FokI	TT	Ref		
	TC	15.870 [0.873-288.501]	6.76	0.00932^∗∗∗^
	CC	26.651 [1.509-470.635]	10.98	0.00092^∗∗∗^
	**Allele frequency difference**			
	T	Ref		
	C	4.574 [1.916-10.922]	13.37	0.00026^∗∗∗^
VDR ApaI	GG	Ref		
	GT	3.357 [0.852-13.229]	3.27	0.0705
	TT	9.641 [2.234-41.603]	11.64	0.00065^∗∗∗^
	**Allele frequency difference**			
	G	Ref		
	T	3.333 [1.650-6.734]	12.06	0.00051^∗∗∗^
VDR TaqI	TT	Ref		
	TC	2.434 [0.701-8.452]	2.05	0.152
	CC	3.265 [0.814-13.099]	2.98	0.084
	**Allele frequency difference**			
	T	Ref		
	C	1.901 [0.954-3.789]	3.4	0.06


*^∗∗∗^*p* < 0.001.*

**Table 3:** Blood lead levels in the study population.

		Normal	High	*p* value
VDR FokI	TC	4.48	10.78	<0.05^∗^
	CC	3.99	18.42	<0.0001^∗∗∗^
VDR ApaI	GG	3.78	15.26	<0.0001^∗∗∗^
	TT	4.59	12.17	<0.05^∗^
VDR TaqI	TT	3.87	18.75	<0.0001^∗∗∗^
	TC	4.15	15.18	<0.01^∗∗^
	CC	4.57	13.43	<0.05^∗^


*^∗^*p* < 0.05; ^∗∗^*p* < 0.01; ^∗∗∗^*p* < 0.001.*

A multiple regression analysis was performed to understand the relationship between various confounders and BLLs. Older age subjects (51–60 years) were 48% more likely to be having higher BLLs than other age group subjects (F statistic: 9.327; *p* = 0.012). Although, the other age groups did not show a significant correlation, overall, there was a 10.4% weak but direct association between age and BLLs. Regression analyses demonstrated that only age made a significant contribution to the prediction of lead levels while other confounding factors were not significant predictors (Table 4).

**Table 4:** Results of multiple regression analyses.

Age (years)	F-statistic	R^2^	Adjusted R	Multiple correlation (R)	*p*-value	Interpretation
21–30	2.2899	0.1252	0.07053	0.3538	0.1497	12.5% association; weak direct relationship
31–40	2.5322	0.1532	0.09268	0.3914	0.1339	15.3% association; weak direct relationship
41–50	3.1402	0.1946	0.1326	0.4411	0.09981	19.5% association; moderate direct relationship
51–60	9.3274	0.4826	0.4309	0.6947	0.01217^∗∗^	48.3% association; strong direct relationship
Overall	3.7147	0.1040	0.07601	0.3225	0.06285	10.4% association; weak direct relationship


*^∗∗^*p* < 0.01.*

## Discussion

Toxicogenetic studies are aimed to understand gene polymorphisms can impact the response of individuals to xenobiotics (Ingelman-Sundberg, 2001) including heavy metals such as lead. The current study was aimed to investigate the association between BLLs and VDR gene polymorphisms at 3 restriction sites namely FokI, ApaI, and TaqI. The target population included in this study are Saudi subjects employed in professions using heavy metal lead like battery workers, plumbers, painters, and vehicle mechanics from Riyadh, Saudi Arabia. Administrative personnel employed at the same places were also chosen to check for BLLs to assess for indirect exposure to lead. The absence of similar studies from this region especially with emphasis on VDR gene in association with heavy metal exposure in an occupational setting will add light on the gravity of xenobiotic exposure in the Saudi population. This is more important owing to the unique ethnicity and socio-demographic characteristics prevalent in the Arab populations.

For the purpose of this study, subjects were categorized into two BLLs sub-groups of <10 μg/dL (Lower BLL group) or >10 μg/dL (Higher BLL group) in accordance with the CDC guidelines (CDC, 1991). A large number of subjects had BLL levels of 4.37 μg/dL, but, a significant proportion of subjects showed BLLs of 18.12 μg/dL (*p* < 0.001). Most subjects working in administrative roles were found to have minimal (<1 μg/dL)/undetectable levels and were excluded from analyses.

To our knowledge, this is a novel and first study to evaluate the possible effects of VDR FokI, ApaI, and TaqI polymorphisms with regards to BLLs in lead exposed workers form Riyadh, Saudi Arabia. The VDR FokI genotypes deviated from the Hardy–Weinberg equilibrium in the overall study population as well as the low BLL group; however, the ApaI and TaqI polymorphisms existed in equilibrium. VDR-Fok1 polymorphism was found to be an independent variable to affect BLLs because it can affect the amino acid sequence in the VDR protein and thus can modify Pb toxicity (Rezende et al., 2008). The VDR FF genotype subjects were reported to have higher BLLs compared with either the heterozygote (Ff) or homozygotes (ff) (Lanphear et al., 2002). In the current study, the VDR CC genotype for FokI polymorphism, VDR TT genotype for ApaI polymorphism, and VDR CC genotype for TaqI polymorphism were found to be in higher percentages in High BLL group subjects than Normal BLL group. The VDR FokI TC, and CC, VDR ApaI GG, and TT genotypes and VDR TaqI TT, TC, and CC in High BLL group showed significantly elevated levels of BLLs compared to normal subjects. A clear association between VDR gene polymorphisms and BLLs has been established in the Saudi Arabian population for the first time.

At a molecular level, evidence indicates that VDR is a DNA-binding transcription factor of the nuclear receptor superfamily (Carlberg and Campbell, 2013). Activation by vitamin D causes heterodimerization with retinoid X receptor which is essential for DNA binding, nuclear translocation, and transcriptional activation and/or suppression (van Etten et al., 2007). The FokI restriction site is located in the exon 2 of the VDR gene which causes an alteration in the start codon (a T/C transition polymorphism [ATG to ACG]) and consequently a VDR protein that is shorter by three amino acids (Whitfield et al., 2001). This can cause altered activity and modification of mineral homeostasis and the regulation of calcium absorption and resorption. While the FokI polymorphism is at the NH2-terminus, where the highly conserved DNA binding domain (DBD) is located, the ligand-binding domain (LBD) is at the variable COOH-terminus of the VDR molecule (Haussler et al., 2013) indicating independent action of each domain. However, the LBD and DBD interact allosterically to regulate VDR gene expression (Orlov et al., 2012). Because the FokI region is located in the DBD, it may cause alterations in the molecular mechanisms that can eventually determine the extent of biological effects caused by high BLLs.

In the context of the current study, it can be hypothesized that the variation in lead absorption may be linked to genetic factors that may influence mineral metabolism (Christakos et al., 2015). Because lead is also a bivalent cation; like calcium, it can compete for absorptive and protein-binding sites (Godwin, 2001), and cellular lead uptake and its toxicokinetics can increase when calcium stores are reduced or depleted. Since calcium metabolism is regulated by VDR, the VDR-Fok1 genotype CC (FF) can be associated with increased bone mineral density and calcium absorption and hence can modify BLLs. This allele has been shown to be a marker for increased calcium absorption compared with the TC (Ff) or TT (ff) genotypes (Haynes et al., 2003) implying that subjects with CC genotype have higher BLL owing to the lead’s chemical similarity with calcium. It is crucial, therefore, that further research is conducted to assess the extent of interactions with respect to calcium metabolism, the FokI polymorphisms and BLLs.

The genotypes TC and CC of the VDR FokI and the TT genotype of VDR ApaI significantly increased the risk of lead toxicity. In addition, the “C” allele of the VDR FokI and “T” allele of the VDR ApaI were found to be high risk alleles. However, the VDR TaqI was not associated with susceptibility to lead toxicity in this population. Regardless of the genotypes, all subjects from high BLL group had considerably increased BLLs compared with Low BLL group.

A study conducted by Haynes et al., 2003 indicated that VDR FokI FF genotype was predominant in the African American population which had the homozygote recessive genotype constituting only 4% of the population. The non-African American and white populations predominantly had the heterozygous genotype (47% and 49%, respectively). The TaqI TT genotype was predominant in this population (89%). A US general population based showed that the Taq1 CC genotype had a negative association with BLLs, while CT or TT had a positive association (Krieg et al., 2010). Research from Saudi Arabia in lean population indicates that the genotype frequencies of VDR ApaI homozygous dominant, heterozygous and homozygous recessive genotypes were 34, 44, and 22%, respectively. The same study indicated that the frequency of VDR TaqI TT, Tt and tt genotypes were 42, 40, and 18%, respectively (Al-Hazmi et al., 2017). These results corroborate our study results regarding the frequency of genotypes in the study population.

The current study also assessed the association between various confounders and their link with BLLs. Older age (51–60 years) was found to be significant predictor for BLLs with 48% strong association (*p* = 0.01). This trend can also be observed in the age group 41–50 years where a 19% moderate association was found between age and BLLs albeit not significant (*p* = 0.09). While age was found to be an important determinant, the duration of work years was not a significant predictor for higher age group indicating that other factors such as overall health status including calcium depletion with age play a major role in determining the effect of lead toxicity; these aspects need further evaluation.

While a study reported that VDR genotypes do not affect long term BLLs (Chuang et al., 2004); our study shows a relation between various genotypes. This could be either due to sample sizes or because the study was conducted over a short term. Long term follow-up studies are needed to conclusively establish the role of VDR FokI, ApaI, and TaqI polymorphisms in lead toxicity. However, the current study demonstrated the role of gene polymorphisms in influencing the susceptibility to lead toxicity and throws a light on the need for further studies especially from Saudi Arabia which constitutes a population with a unique ethnicity and genetic makeup. Identifying the genetic markers to understand the toxic effects caused by xenobiotic exposure among high-risk individuals may provide a tool for primary prevention, and suggest mechanisms for intervention. In several previous studies, large inter-ethnic differences in the frequencies of different gene variants have been reported especially with reference to the VDR gene (HSDB, 2007). Owing to the critical role of VDR in calcium metabolism and the structural similarity of Pb2+ ions to mimic and replace Ca2+ ions, there is an urgent need to include toxicogenetic approaches for accurate determination of heavy metal toxicity.

## Ethics Statement

This study was carried out in accordance with the recommendations of “Institutional Ethics Review Board, College of Medicine and King Khalid University Hospital, King Saud University, Riyadh, Saudi Arabia” with written informed consent from all subjects. All subjects gave written informed consent in accordance with the Declaration of Helsinki. The protocol was approved by the “Institutional Ethics Review Board College of Medicine and King Khalid University Hospital, King Saud University, Riyadh, Saudi Arabia”.

## Author Contributions

All authors have contributed to the conceptualization, design, methodology, data collection, results interpretation, and writing the manuscript.

## Conflict of Interest Statement

The authors declare that the research was conducted in the absence of any commercial or financial relationships that could be construed as a potential conflict of interest.
