# Supplementary material for: Vitamin D Receptor FokI, ApaI, and TaqI Polymorphisms in Lead Exposed Subjects From Saudi Arabia
Source: Front Genet. 2019 Apr 26;10:388. doi: 10.3389/fgene.2019.00388 (PMC6497804; doi:10.3389/fgene.2019.00388)

**Vitamin D receptor (VDR), 12q13.11, NM_001017535.1; Fragment-1:**Top of Form\

GCCCACCCTTGCTGAGCTCCCTGGTGGTGGGGGGTGGGGGCGGTGGGATGAGGCTGGGGG

TGGGTGGCACCAAGGATGCCAGCTGGCCCTGGCACTGACTCTGGCTCTGACCGTGGCCTG

CTTGCTGTTCTTACAGgg**atg**↓gaggcaatggcggccagcacttccctgcctgaccctgga gactttgaccggaacgtgccccggatctgtggggtgtgtggagaccgagccactggcttt

cacttcaatgctatgacctgtgaaggctgcaaaggcttcttcaggtgagccctcctccca

ggctctccccagtggaaagggagggagaagaagcaaggtgtttccatgaagggagccctt VDR-Ex2-R

**Fragment-1:**

1. **VDR-Ex2-F GAGCTCCCTGGTGGTGG**

**VDR-Ex2-R CCCTTCATGGAAACACCTTG**

**FokI**: (GGATG/)

WILD TYPE: TT: 2 FRAGMENTS OF 128+212 bp

HETEROZYGOUS: TC: 3 FRAGMENTS, 128+212+341 bp

HOMOZYGOUS MUTANT CC: 1 FRAGMENT, 341 bp


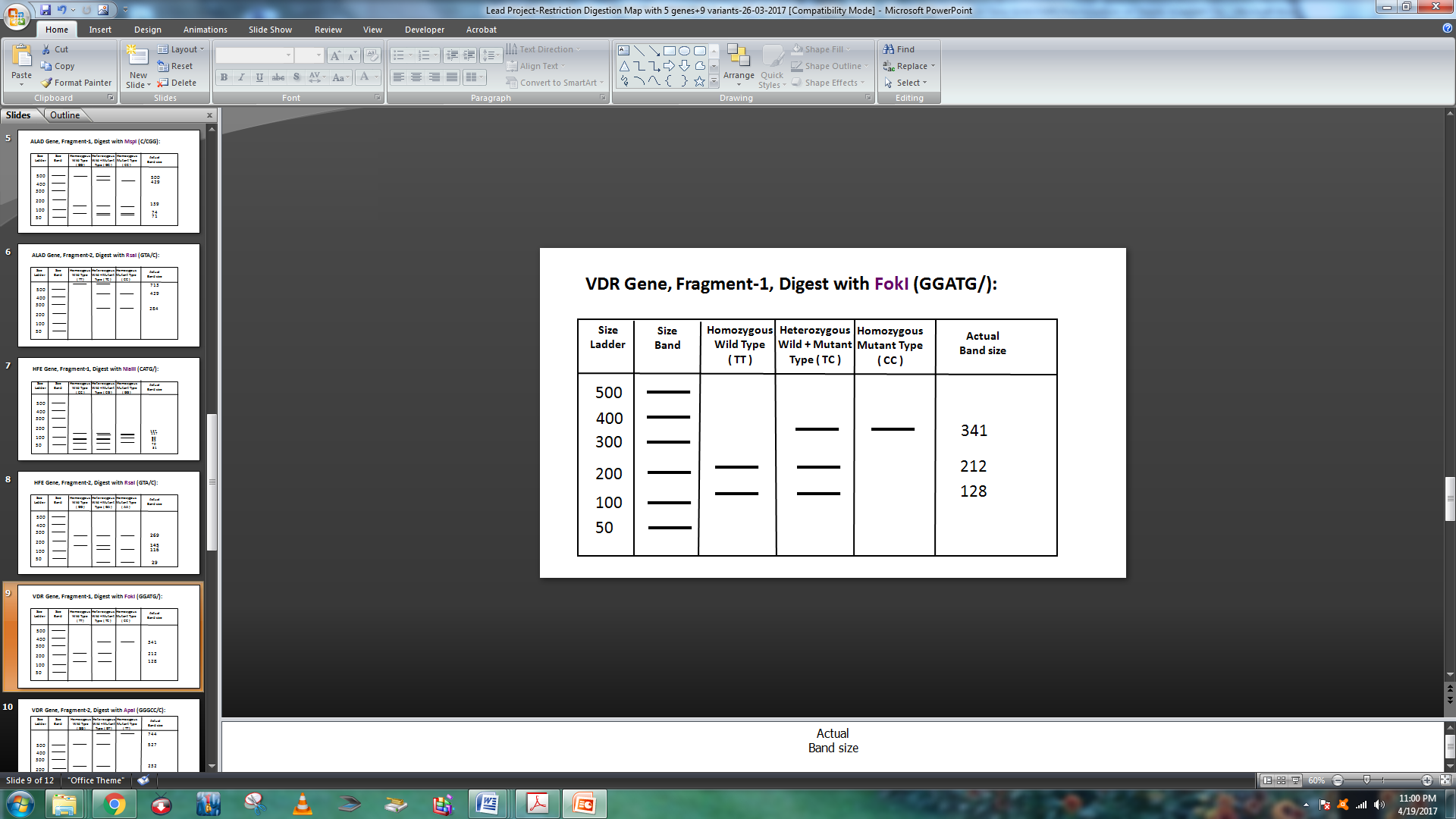


**Vitamin D receptor (VDR), 12q13.11, NM_001017535.1; Fragment-2:**Top of Form\

CGCATCGGGACGCTGAGGGATGGACAGAGCATGGACAGGGAGCAAGGCCAGGCAGGGACA VDR-Int8-F2

GGGCCAGGTGCGCCCATGGAAGGACCTAGGTCTGGATCCTAAATGCACGGAGAAGTCACT

GGAGGGCTTTGGGGCCAGGCAGTGGTATCACCGGTCAGCAGTCATAGAGGGGTGGCCTAG

GGGGTGCTGCCGTTGAGTGTCTGTGTGGGTGGGGGGTGGTGGGATTGAGCAGTGAGGGGC **ApaI**

C↓CAGCTGAGAGCTCCTGTGCCTTCTTCTCTATCCCCGTGCCCACAGatcgtcctggggtg

Caggacgccgcgctgatt↓gaggccatccaggaccgcctgtccaacacactgcagacgtac **TaqI**

atccgctgccgccacccgcccccgggcagccacctgctctatgccaagatgatccagaag

ctagccgacctgcgcagcctcaatgaggagcactccaagcagtaccgctgcctctccttc

cagcctgagtgcagcatgaagctaacgccccttgtgctcgaagtgtttggcaatgagatc

tcc**tga**ctaggacagcctgtggcggtgcctgggtggggctgctcctccagggccacgtgc

caggcccggggctggcggctactcagcagccctcctcaccccgtctggggttcagcccct

cctctgccacctcccctatccacccagcccattctctctcctgtccaacctaaccccttt

cctgcgggcttttccccggtcccttgagacctcagccatgaggagttgctgtttgtttga VDR-Int8-R2

**Fragment-2:**

**VDR-Int8-F2 CAGAGCATGGACAGGGAGCAAG**

**VDR-Int8-R2 CAACTCCTCATGGCTGAGGTCTC**

**ApaI**: (GGGCC/C)

WILD TYPE: GG: 2 FRAGMENTS OF 232+527 bp

HETEROZYGOUS: GT: 3 FRAGMENTS, 232+527+744 bp

HOMOZYGOUS MUTANT TT: 1 FRAGMENT, 744 bp no cut


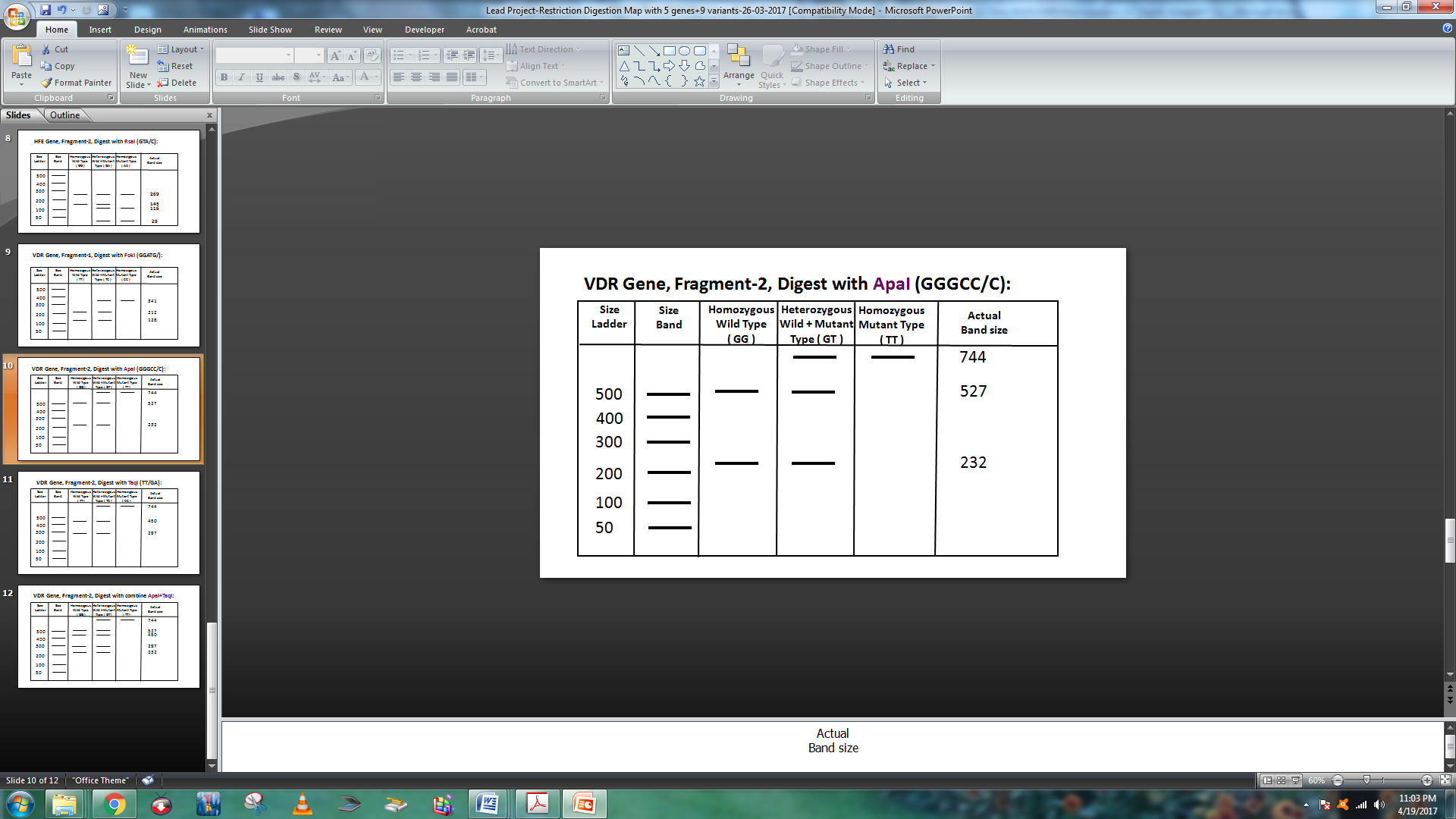
**Vitamin D receptor (VDR), 12q13.11, NM_001017535.1; Fragment-3:**Top of Form\

CGCATCGGGACGCTGAGGGATGGACAGAGCATGGACAGGGAGCAAGGCCAGGCAGGGACA VDR-Int8-F2

GGGCCAGGTGCGCCCATGGAAGGACCTAGGTCTGGATCCTAAATGCACGGAGAAGTCACT

GGAGGGCTTTGGGGCCAGGCAGTGGTATCACCGGTCAGCAGTCATAGAGGGGTGGCCTAG

GGGGTGCTGCCGTTGAGTGTCTGTGTGGGTGGGGGGTGGTGGGATTGAGCAGTGAGGGGC **ApaI**

C↓CAGCTGAGAGCTCCTGTGCCTTCTTCTCTATCCCCGTGCCCACAGatcgtcctggggtg

Caggacgccgcgctgatt↓gaggccatccaggaccgcctgtccaacacactgcagacgtac **TaqI**

atccgctgccgccacccgcccccgggcagccacctgctctatgccaagatgatccagaag

ctagccgacctgcgcagcctcaatgaggagcactccaagcagtaccgctgcctctccttc

cagcctgagtgcagcatgaagctaacgccccttgtgctcgaagtgtttggcaatgagatc

tcc**tga**ctaggacagcctgtggcggtgcctgggtggggctgctcctccagggccacgtgc

caggcccggggctggcggctactcagcagccctcctcaccccgtctggggttcagcccct

cctctgccacctcccctatccacccagcccattctctctcctgtccaacctaaccccttt

cctgcgggcttttccccggtcccttgagacctcagccatgaggagttgctgtttgtttga VDR-Int8-R2

**Fragment-3:**

**TaqI**: (TT/GA)

WILD TYPE: TT: 2 FRAGMENTS OF 297+450 bp

HETEROZYGOUS: TC: 3 FRAGMENTS, 297+450+744 bp

HOMOZYGOUS MUTANT CC: 1 FRAGMENT, 744 bp no cut


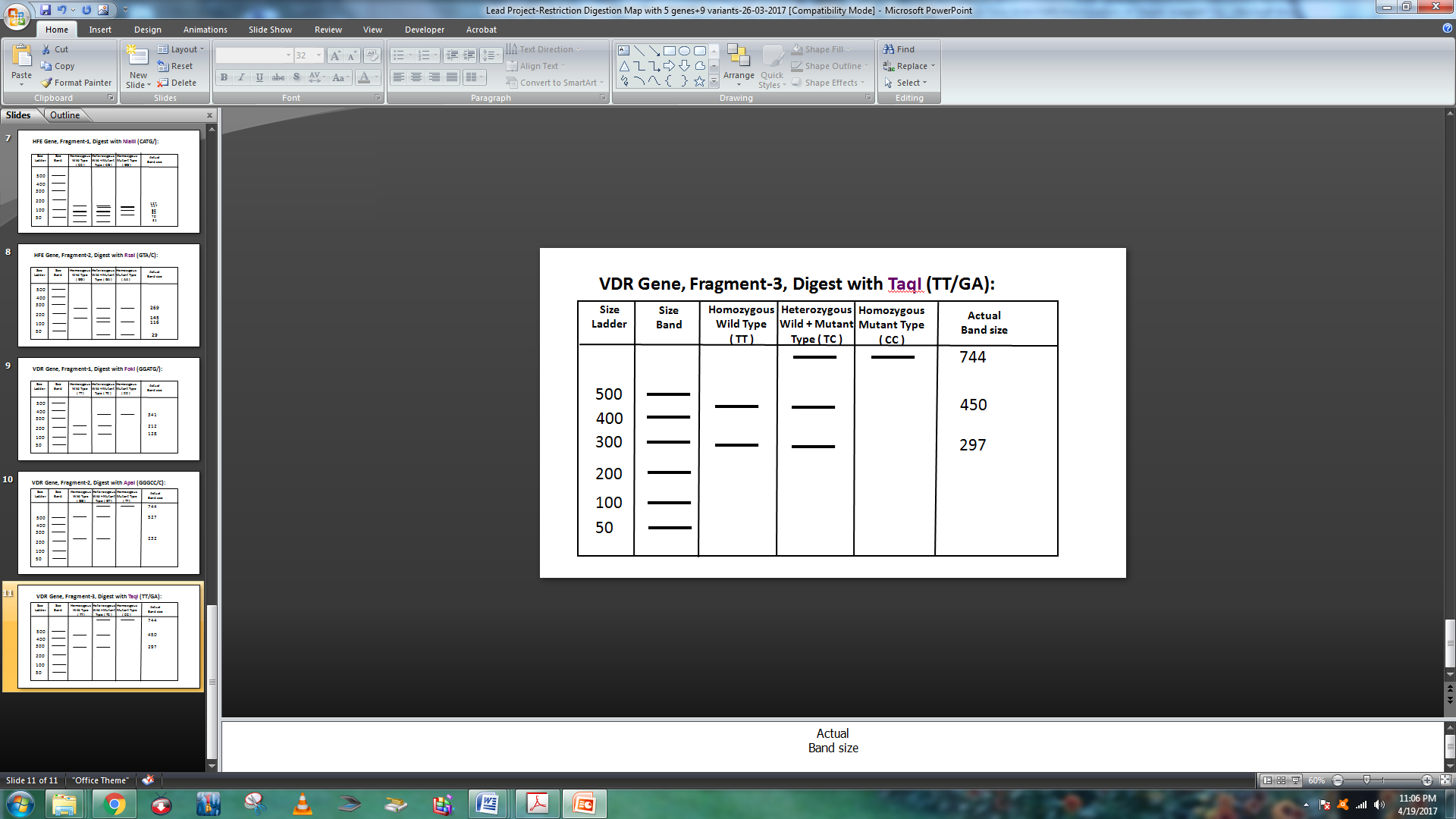

Supplement: Supplementary file 1 [file Table_1.DOCX]
